# Case report: A rare cause of intestinal perforation in a third-trimester pregnant woman

**DOI:** 10.3389/fmed.2024.1387043

**Published:** 2024-07-03

**Authors:** Clemens Stiegler, Christopher Kapitza, Florian Weber, Wladimir Patalakh, Claus Schäfer

**Affiliations:** ^1^Medical Department II, Klinikum Neumarkt, Neumarkt in der Oberpfalz, Germany; ^2^Institute of Pathology, University of Regensburg, Regensburg, Germany; ^3^Department for Surgery, Klinikum Neumarkt, Neumarkt in der Oberpfalz, Germany

**Keywords:** endometriosis, intestinal perforation, appendicitis, peritonitis, sonography, point-of-care ultrasound, POCUS, pregnancy

## Abstract

**Background:**

An acute abdomen is a medical emergency that requires early diagnosis and treatment. In pregnancy, this process is significantly more challenging, and radiological findings are sometimes unclear due to the enlarged uterus displacing other structures. Moreover, endometriosis-related complications are rare, and the disease is often undiagnosed.

**Case presentation:**

We report a case of acute perforation of the cecum and appendix during pregnancy (35 weeks of gestation) caused by a previously unknown, deep infiltrating endometriosis with focal ulceration of the affected bowel wall, which sonographically seemed to be acute appendicitis.

**Conclusion:**

Despite the relatively low risk, clinicians should be aware of possible endometriosis-associated complications in pregnancy with potentially life-threatening events, even in previously unknown endometriosis. Further studies should evaluate intestinal complications during pregnancy in relation to previous treatment of intestinal endometriosis (conservative vs. surgical).

## Introduction

Endometriosis, defined as the extrauterine occurrence of endometrial tissue, mainly affects women of fertile age, with a prevalence of approximately 10% (1). The median interval between diagnosis and onset of symptoms is approximately 10 years, so the number of unreported patients with endometriosis is likely higher, especially in extragenital cases (2). Correspondingly, incorrect diagnoses are made frequently. Approximately 12–15% of patients with endometriosis have intestinal involvement, with the rectosigmoid being the predilection site in the GI tract due to its proximity to the uterus (in 50–90% of cases), while the appendix (3–18%) and the cecum (2–5%) are affected less frequently (3).

Acute onset, right-sided lower abdominal pain during pregnancy is a challenge for diagnostics and requires rapid clarification. Regarding the initial diagnostic workup, sonography plays a crucial role. The German guideline for the treatment of acute appendicitis in adults, as well as the European Federation for Ultrasound in Medicine and Biology (EFSUMB) position paper, both recommend ultrasound as the primary imaging method, with a sensitivity of 71–94% and a specificity of 81–98%, especially since the rate of false-positive appendectomies can be significantly reduced by sonography (4–6). Here, we present a rare case of cecum and appendix perforation in a pregnant patient in the third trimester caused by deep infiltrating endometriosis.

## Case presentation

A 37-year-old woman (second-gravida) in her 35th week of pregnancy (34 + 5) was admitted to our hospital with intense right-sided lower abdominal pain and increased inflammatory markers (CRP 156 mg/L, leukocytes 17/nl). The pregnancy had been inconspicuous up to that point. The patient noted stool irregularities that had previously occurred before pregnancy. A colonoscopy, which was performed 2 years ago, showed no significant findings. There were no known preexisting diseases. The patient had been experiencing abdominal pain for 2 days, as well as nausea, emesis, and fever.

**Figure 1:**
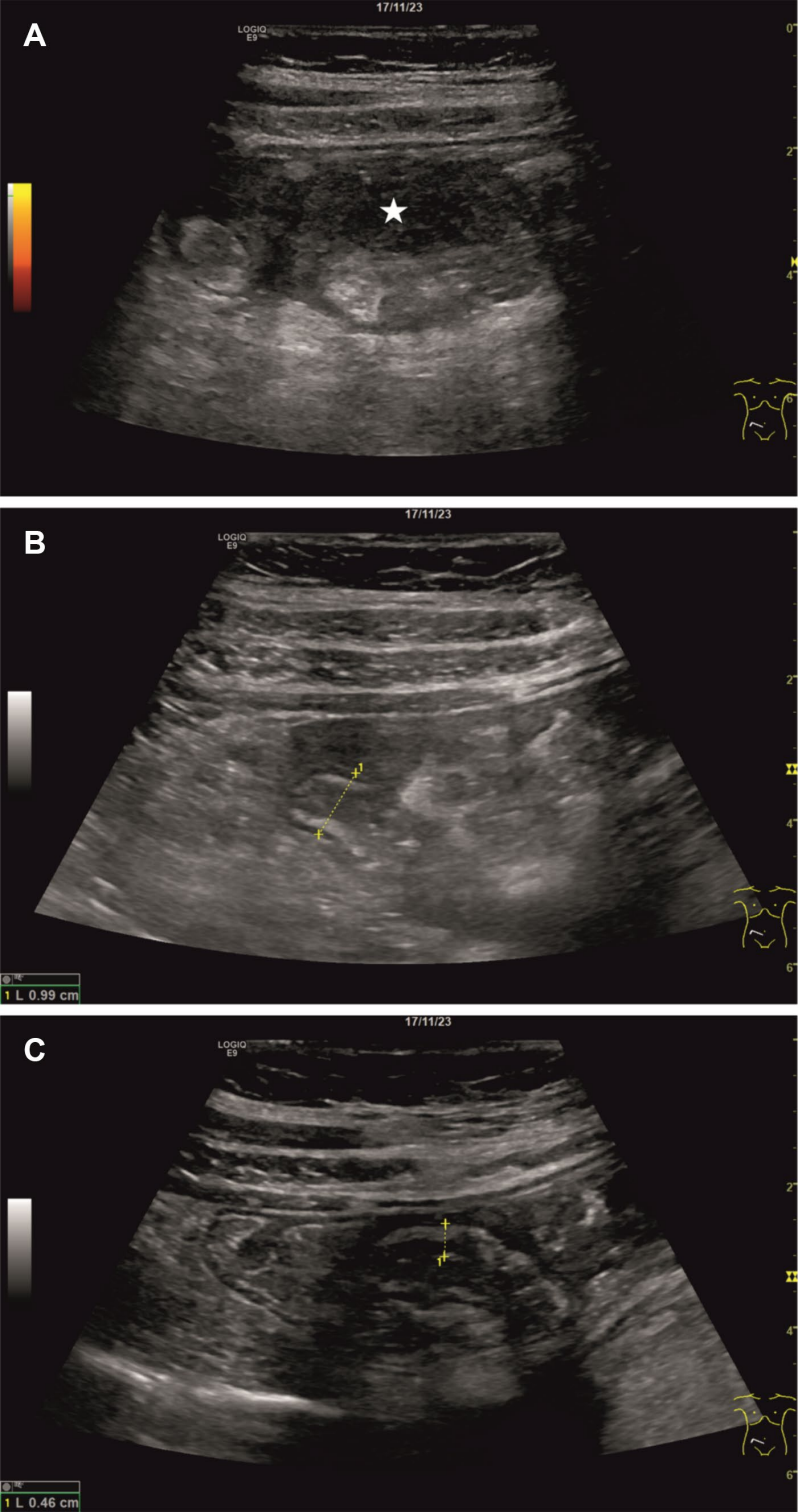
Results of the sonographic examination. A small amount of hypoechoic and echo-inhomogeneous free peritoneal fluid could be detected between the cecum and the uterus (**A**; *white star*). Moreover, a structure with a diameter of approximately 1 cm and a thickened, hypoechoic wall was found – most likely corresponding to the vermiform appendix **(B)**. Additionally, the walls of the cecum and the terminal ileum appeared hypoechoic and thickened **(C)**.

The physical examination revealed tenderness on palpation in the right lower abdomen and flank pain. There was no rebound tenderness and no muscular guarding. The gynecological examination was unobtrusive, the pregnancy was intact, the CTG was inconspicuous, and the patient’s vital signs (RR 140/90 mmHg, HR 83/min, SpO_2_ 96%). Due to the limited examination conditions, the initial emergency ultrasound showed no significant results. As expected, the uterus appeared widely extended in the abdomen, causing a lateral displacement of the intestinal loops, which made the sonographic assessment even more complicated. The gallbladder showed no sonomorphological abnormalities, ruling out acute cholecystitis. Moreover, no appendiceal dilatation and no free peritoneal fluid could be detected. A right-sided hydronephrosis was considered physiological during pregnancy. Since no leukocyturia was detected in disposable catheter urine, a urinary tract infection could also be excluded. However, inflammatory markers showed a further increase (CRP 193 mg/L) within 1 day, so gynecological and gastroenterological examinations were repeated the next day. There were no gynecological changes. In contrast, a recent ultrasound examination revealed a small quantity of echo-poor and echo-inhomogeneous free peritoneal fluid between the cecum and the uterus (Figure 1A). While the ascending colon appeared normal, a slight wall thickening of the cecum and the terminal ileum could be found (Figure 1C). Echogenicity of adjacent mesenterial adipose tissue was increased, most likely because of an ongoing inflammatory process. After prolonged searching, a tubular structure with a diameter of approximately 1 cm and a thickened, hypoechoic wall of unknown origin was found (Figure 1B). In summary, we postulated the preliminary diagnosis of acute appendicitis with a concomitant inflammatory reaction of the adjacent intestinal loops and inflammatory altered ascites in the right lower abdomen.

**Figure 2:**
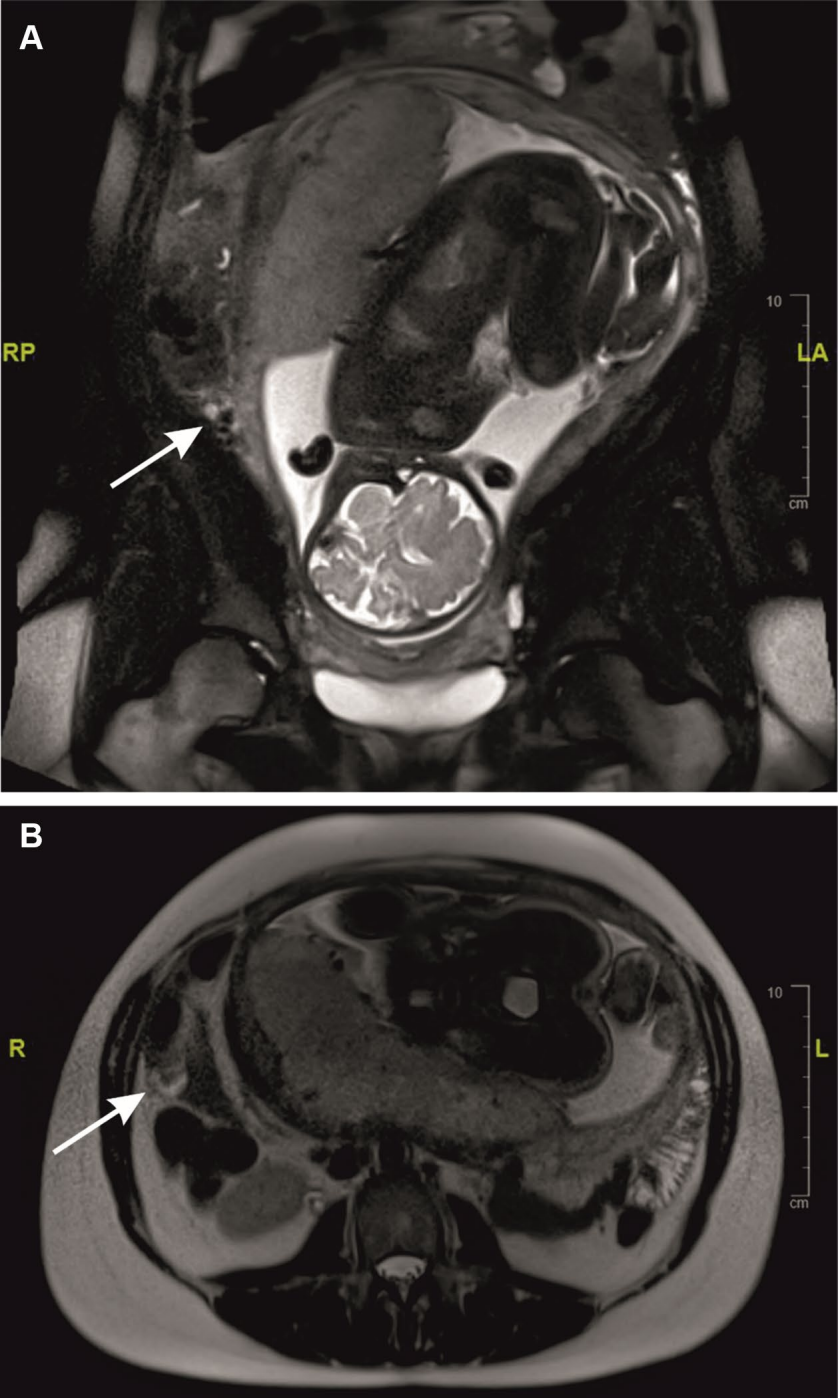
MRI examination. A small margin of free peritoneal fluid was found at the presumed base of the appendix (**A,B**; *white arrows*).

**Figure 3:**
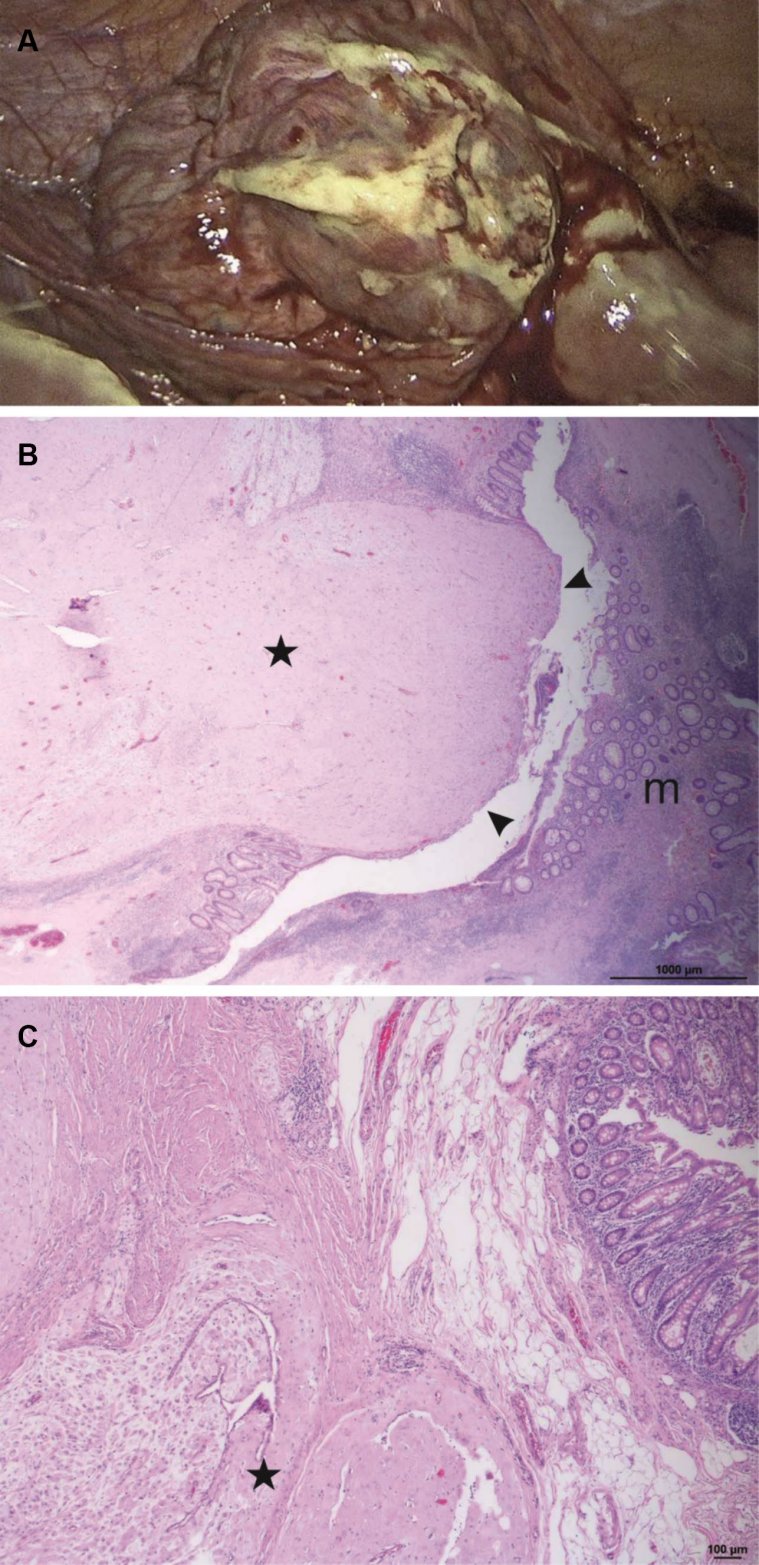
Laparoscopic and histopathological findings. The vermiform appendix showed a bulgy thickening at the apex and the walls of the cecum and the appendix were perforated **(A)**. Apex of the vermiform appendix (H&E, 25x) with masses of decidualized endometrium (*star*) comprising the entire wall and perforating into the lumen of the appendix (*arrowheads*), leading to perforation due to gestational increase in volume. Normal mucosa on the right side (*m*) **(B)**. Higher magnification (H&E, 50x) shows decidualized stroma with myxoid changes and endometrioid glands (*star*) within the wall of the appendix **(C)**.

As a next step, our surgical colleagues arranged an additional magnetic resonance tomography (MRI) examination (Figure 2), in which the vermiform appendix could not be identified. A small margin of free peritoneal fluid was found at the presumed base of the appendix (Figure 2, white arrows), which underlined an irritative process in this region. No evidence of a significant phlegmonous penetration or infiltration in the right lower abdomen could be found. The findings of these sonographic and MRI examinations indicated that surgical treatment was necessary. Intraoperatively, fibrinous peritonitis in all four abdominal quadrants was found. The vermiform appendix showed a bulgy thickening at the tip, and the cecum and appendix were perforated (Figure 3A). Consequently, a laparoscopic resection of the cecal pole was performed. The histopathological examination of the resection revealed putrid, melting foci up to 1 cm in width in the subserosa. Formations of decidualized endometrium with partly myxoid loosening and variously sized glands, which were lined by cubic epithelium, were found both at the appendiceal apex and in the resected cecal parts (Figures 3B,C). One of the largest decidua complexes appeared within the wall of the apex of the vermiform appendix, leading to subsequent perforation (Figure 3B).

In summary, an extensive, tumorous endometriosis extragenitalis with decidualization during pregnancy and thus a strong progression of size led to the destruction of the walls of both the appendix vermiformis and the cecum, which consecutively resulted in a fibrinous-purulent peritonitis.

Six days after surgery and supplementary intravenous antibiotic therapy, the patient could be discharged from the hospital with decreasing inflammatory parameters and an intact pregnancy. Four weeks later, a healthy boy (weight 2,820 g, body length 52 cm, APGAR 9/10/10) was delivered by caesarean section. There were lesions of endometriosis at the anterior wall of the uterus, which were coagulated due to minor bleeding. We recommended that the patient undergo a colonoscopy in order to detect any remaining endometriosis sites in the colon.

## Discussion

Endometriosis can be differentiated between the intrapelvic variant, which affects structures in the small pelvis, and the rare extrapelvic or extragenital manifestation, in which the gastrointestinal (GI) tract and the urogenital tract are most frequently affected (7, 8). Moreover, intrapelvic endometriosis can be divided into a superficial peritoneal and a deep infiltrating form (infiltration of at least 5 mm).

Although different theories on the pathogenesis of endometriosis exist, the exact mechanism is not yet fully understood. Most likely, there is an interaction of different factors. It is well described that women with increased retrograde menstruation, e.g., due to outflow obstruction, have a higher prevalence of endometriosis. On the other hand, patients with endometriosis exhibit a higher number of endometrial progenitor cells during menses. However, the importance of mesenchymal stem cells, which are recruited by endometriosis lesions using the C-X-C motif chemokine ligand 12/14 (CXCL12/CXCR14) signaling pathway, is currently under discussion. Investigations revealed that CXCL12 is expressed four to six times more frequently in endometriosis lesions than in eutopic endometrium (9). Moreover, the expression of CXCL12 and C-X-C motif chemokine receptor 4 (CXCR4) is also estrogen-dependent (10). Sprawling localizations, such as in the thorax or brain, can be explained by an assumed hematogenic dissemination of stem cells (7).

Endometriosis can be associated with dysmenorrhea, dyspareunia, pelvic pain, and infertility. Moreover, GI involvement is often accompanied by cycle-related abdominal cramps, meteorism, stool irregularities such as diarrhea or constipation, nausea and vomiting, or perianal bleeding. In rare cases, finally, even intestinal obstruction can occur (7, 11, 12). If the ileum is the main area of manifestation, the resulting symptoms can imitate an active Crohn’s disease and therefore complicate the diagnostic procedures (13, 14). In addition, deep infiltrating endometriosis is significantly more common in patients with chronic inflammatory bowel diseases (CIBDs) (15). However, in case of sudden onset of pain in the right lower abdomen in combination with local muscular guarding and positive clinical signs typical of appendicitis, e.g., the psoas sign or Rovsing’s sign, endometriosis may deceptively mimic acute appendicitis, as presented in our case report (12). A hemoperitoneum due to endometriosis is an important differential diagnosis of acute appendicitis in cases of sudden onset of lower abdominal pain during pregnancy and can lead to significant blood loss resulting in a hypovolemic shock, which puts mother and child at risk (16, 17). In our patient, the stability of the hemoglobin level and stable vital signs ruled out this complication. Nevertheless, acute appendicitis is one of the most common causes of right-sided lower abdominal pain in pregnant women, with a high risk for a complicated progression with abscessation and a high fetal mortality (18). Regarding the initial diagnostic workup of right-sided lower abdominal pain, sonography plays a crucial role, as mentioned above. The diagnostic accuracy as well as the sensitivity and specificity of high-end ultrasound devices are now higher than 90% and thus at the level of computed tomography (CT) and MRI, although the result depends on the sonographer’s experience (4). Furthermore, for point-of-care ultrasound (POCUS), sensitivity and specificity of more than 90% were shown, so a primary ultrasound should be performed in the emergency room in order to set the course for the further treatment path (19). In case of initial sonomorphologically unclear results, repeated sonography may increase the diagnostic accuracy (20).

Although classical acute appendicitis was not present in our case, corresponding pathognomonic signs such as the sonographic target sign, the pressure pain in the right lower abdomen, and hyperechogenic tissue alongside an echo-complex fluid accumulation could be found as essential sonographic diagnostic criteria of acute appendicitis (4). The non-homogeneous echo pattern of the abdominal fluid accumulation suggested a complicated process with abscessation. In our case, the perforation of the appendix resulted in purulent peritonitis due to endometriosis, which finally produced the same sonographic picture as in primary appendicitis with perforation and abscess formation.

In unclear cases, MRI is recommended in pregnant women as the imaging method of choice for suspected acute appendicitis. Sensitivity and specificity are higher in this special patient collective than in ultrasound, with 80 to 86% and 97 to 99%, respectively (21–24). The sonographic accuracy decreases after the first trimester owing to the difficult delineation of the appendix (25).

Although the overall diagnostic accuracy of the method is high even in pregnant women, it is not clear which examination protocols are preferable and which are less accurate (26). The unclear MRI findings in our patient reflect the greater challenges faced by imaging techniques in the third trimester, as the appendix is often further cranially and atypically located because of the expansion of the uterus (21). As described in other case reports, in advanced pregnancy, the appendix can often not be reliably identified by cross-sectional imaging (MRI, CT) (27, 28). Therefore, clinical and laboratory parameters play an important role in a possible indication of a laparoscopic intervention.

Endometriosis was not previously known in our patient, and there were no problems in her first pregnancy. However, as her wish to have more children remained unfulfilled for 2 years, endometriosis was suspected by the gynecologists but not diagnosed. This observation is consistent with descriptions in other case reports, where endometriosis is unknown. Therefore, it can be assumed the disease is significantly underdiagnosed (11–13, 17, 29, 30). It is well known that endometriosis can have adverse effects on pregnancy, causing higher rates of abortions and premature births, decreased birth weight of the child, gestational diabetes, and hypertensive pregnancy disorders (31, 32). However, intestinal perforation caused by endometriosis is a rare event in pregnancy and usually occurs in the third trimester. As previously mentioned, there are different theories on the pathogenesis of endometriosis, but so far, especially the pathomechanisms of intestinal perforation caused by endometriosis, remain unclear. In general, endometriosis improves during pregnancy (29, 30, 33). At least in superficial endometriosis, decidualization by hormone influence in pregnancy prevents progression, which is therapeutically addressed by the intake of hormone preparations in non-pregnant women. Whether deep-infiltrating endometriosis reacts differently to hormonal influences is not clear (29). Size regulation under the influence of progesterone can lead to the weakening of the intestinal wall so that, in combination with fibrosis and adhesions, perforation can be promoted (28, 29). It is also discussed that the so-called stromal endometriosis, a histological subtype lacking glands and spiral arteries, is rather asymptomatic in non-pregnant women and can lead to growth and thus to perforation during pregnancy under the influence of hormones (27). In the present case, we consider the absence of the stromal variant since glands were confirmed histologically.

## Conclusion

This case shows a rare, severe complication of endometriosis in pregnancy in the third trimester, which presented as perforated appendicitis. Given the difficulties of imaging diagnostics in late pregnancy, we want to emphasize the importance of sonography as the initial diagnostic modality, which does not lead to radiation exposure and therefore is without risk for mother and child. Repeated sonography with high-end equipment and experienced investigators can increase sensitivity and speed up the diagnosis.

## Data availability statement

The original contributions presented in the study are included in the article/supplementary material, further inquiries can be directed to the corresponding author.

## Ethics statement

Written informed consent was obtained from the individual(s) for the publication of any potentially identifiable images or data included in this article.

## Author contributions

CSt: Conceptualization, Investigation, Methodology, Project administration, Resources, Visualization, Writing – original draft, Writing – review & editing. CK: Conceptualization, Methodology, Visualization, Writing – original draft, Writing – review & editing. FW: Methodology, Writing – review & editing. WP: Methodology, Writing – review & editing. CSc: Supervision, Writing – review & editing.
